# Molecular Target and Action Mechanism of Anti-Cancer Agents

**DOI:** 10.3390/ijms24098259

**Published:** 2023-05-04

**Authors:** Seok-Geun Lee

**Affiliations:** 1 Department of Biomedical Science & Technology, Kyung Hee University, Seoul 02447, Republic of Korea; seokgeun@khu.ac.kr; Tel.: +82-2-961-2355; 2 BioNanocomposite Research Center, Kyung Hee University, Seoul 02447, Republic of Korea

Precision oncology, also known as personalized medicine, is an evolving approach to cancer treatment that aims to tailor therapies to individual patients based on their unique molecular profile, including genetic alterations and other biomarkers [1,2,3]. By identifying specific molecular alterations that drive cancer growth and developing drugs that target these alterations, precision oncology aims to achieve more effective and less toxic treatments than conventional chemotherapy. Although precision oncology has proven successful in clinical practice for certain cancer types, such as chronic myeloid leukemia (CML) with the use of imatinib, most cancer patients still rely on traditional strategies of surgical resection, chemotherapy, and radiotherapy [2,3,4]. Nevertheless, precision oncology offers great potential for improving cancer treatment outcomes and enhancing patient quality of life. To achieve this goal, several challenges must be addressed, including the development of new anti-cancer agents that target genetic changes, effective biomarkers to identify patients who are most likely to benefit from targeted therapies, and overcoming resistance to these therapies [2,3].

This Special Issue features 10 research articles and 8 reviews that provide significant insights into anti-cancer agents and their targets and action mechanisms, highlighting the importance of precision oncology in cancer treatment. Some of the research articles report on the discovery of novel compounds. For example, a new series of purine derivatives led to the identification of a novel SMO antagonist compound **4s** that inhibits the Hedgehog signaling pathway, showing anti-cancer effects even better than vismodegib, a well-known SMO antagonist used in the oncology clinic [5]. Another article identified a new multi-kinase inhibitor, 3-[(3-hydroxy-4-methoxy)phenyl]-2-(thien-2-yl)acrylonitrile **1c**, with preferential activity against the VEGFR-2, showing better anti-cancer effects compared to sorafenib in hepatoma cells [6]. Additionally, new chimeric inhibitors targeting both EGFR and HDACs, 3BrQuin-SAHA and 3ClQuin-SAHA, were shown to have pronounced anti-neoplastic effects in both solid and liquid tumor cells [7]. The promising results of these novel compounds warrant further investigations to confirm their anti-cancer effects and detailed mechanisms of action, as well as clinical trials to establish their suitability for use in the clinic.

Imatinib and dasatinib are medications used to treat Philadelphia chromosome-positive CML and acute lymphocytic leukemia (ALL) by targeting aberrant BCR-ABL kinase [8]. Imatinib also inhibits other tyrosine kinases (TKs), such as c-KIT and PDGFR, making it useful for treating other malignancies such as gastrointestinal stromal tumors (GISTs) [8]. Similarly, dasatinib inhibits other TKs, such as PDGFR and EphA, and is being investigated for its potential use in various other malignancies [8]. Two articles in this Special Issue describe novel molecular targets and action mechanisms of these drugs. Increased oxidative phosphorylation by downregulating miR-483-3p was revealed as a part of imatinib’s action mechanism in treating GISTs, while p90RSK was identified as a novel target for dasatinib’s potential use in gastric cancer [9,10]. Understanding the detailed action mechanisms of the drugs can provide valuable evidence and insight into their potential extended use in the clinic.

Natural products and their derivatives are promising sources for developing anti-cancer agents due to their safety and low risk of toxicity compared to synthetic chemical drugs [11,12]. Two articles in this Special Issue explore potential anti-cancer agents from natural products. Ginkgolide C, a phytochemical from *Ginkgo biloba*, exhibited anti-cancer effects in hepatocellular carcinoma cells by inhibiting HGF-induced c-MET activation, while 23-demethyl 8,13-deoxynargenicin, a nargenicin A1 analog, targeted cyclophilin A in gastric cancer cells [13,14]. Further studies are needed to reveal more detailed action mechanisms, develop more derivatives with enhanced activity, and evaluate their potential for use in oncology clinics.

Several articles in this Special Issue propose new targets for developing anti-cancer agents. One article uncovered the novel role of promyelocytic leukemia proteins in regulating the Fanconi anemia pathway and DNA damage-induced CHK1 phosphorylation, suggesting CHK1 as a potential anti-cancer drug target to reduce the DNA repair capacity of cancer cells [15]. Another article suggests USP7 as a promising target for cancer therapy [16]. A comprehensive review article provides insights into the functions of RPS6 in cancer, including its transcriptional regulation, upstream regulators, and extra-ribosomal roles, and finally discusses its potential as a therapeutic target for cancer [17]. In addition, another review discusses the potential of noncoding RNAs, such as microRNAs, and long noncoding RNAs as therapeutic targets for estrogen receptor-positive breast and ovarian cancers [18]. Lastly, a review article discusses the role of RANK and the RANK ligand (RANKL) pathway in breast cancer initiation and progression, as well as its potential as a target for the prevention and treatment of breast cancer [19]. The article introduces denosumab, a monoclonal antibody against RANKL, which has been widely used in breast cancer patients with metastatic diseases [19].

Furthermore, the articles in the Special Issue suggest various therapeutic strategies, including drug combinations to improve therapeutic effects and overcome side effects. For example, one potential strategy discussed is the combination of a RANKL inhibitor, such as denosumab, with immune checkpoint inhibitors, such as anti-CTLA-4 and anti-PD-1 monoclonal antibodies, for breast cancer. The review also discusses recent clinical trials of combining denosumab with various other interventions for breast cancer patients, as well as its combination with checkpoint inhibitors for other solid tumors [19]. Another valuable strategy mentioned is the combination of inhibitors of PLK1 and USP7 for treating paclitaxel-resistant cancers, although further studies are required [16]. In addition, a study showed that vitamin D has a cytoprotective effect on doxorubicin-induced cardiac toxicity in a triple-negative breast cancer (TNBC) mouse model, suggesting a combination of vitamin D and doxorubicin as a promising therapeutic strategy for TNBC to reduce the side effects of doxorubicin, such as cardiotoxicity [20]. The use of various combinations in clinical practice and the continuous development of these strategies indicate that a systemic approach that incorporates all related knowledge is essential for anti-cancer therapy.

This Special Issue also features a review article by Professor Paul B. Fisher, a distinguished oncology scholar who originally isolated MDA-7/IL-24 30 years ago [21]. The article reviews the molecule’s journey from its identification as a tumor suppressor to its potential as a therapeutic molecule for gene therapy, including a phase I clinical trial. The article also explores its combination with other therapeutic modalities such as radiotherapy, cisplatin, celecoxib, and gefitinib [21].

Another review in this issue provides an updated overview of the current standard and experimental therapies for glioblastoma [22]. The paper highlights the molecular characteristics of glioblastoma stem cells (GSCs), which can cause resistance to current treatments. While temozolomide, an alkylating agent, remains the standard treatment for glioblastoma patients, the review proposes a new antitumor strategy for treating GSCs by promoting their differentiation. Additionally, the review emphasizes the importance of targeting GSCs in conjunction with standard radiochemotherapy, which could potentially lead to improved outcomes for patients with glioblastoma [22].

Two other review papers in the Special Issue suggest modifications and improvements to conventional chemotherapeutic drugs, such as cisplatin and its derivatives [23,24]. Although cisplatin has been a common and crucial conventional platinum-based chemotherapy for various types of human cancer since the 1970s, problematic issues with platinum-based anti-cancer drugs, such as low solubility and toxicity, have stimulated the search for alternatives. The reviews propose palladium- and selenium-based compounds as potential anti-cancer agents and introduce their potential combinatorial strategies with other drugs [23,24]. They also discuss recent and current clinical trials of platinum-, palladium-, or selenium-based therapies [23,24].

Despite the continued reliance on traditional chemotherapies for many advanced cancer patients, research efforts have led to successful precision medicine treatments for certain cancer types such as CML and GISTs [2,3,4]. Another review article in this issue provides an updated overview of the current treatment options for GISTs, primarily including TK inhibitors (TKIs), such as imatinib, sunitinib, and regorafenib. The review highlights the issue of pharmacological resistance to TKIs and explores new treatment options, such as ripretinib, avapritinib, and cabozantinib, that have shown promising results in clinical trials [25]. Additionally, the paper discusses the potential of combining immunotherapy with TKIs and provides information on the different mutations that can occur in GISTs and their impact on treatment options [25]. This information is critical for clinicians treating patients with GISTs as it underscores the need for personalized treatment plans based on the specific mutations present in each patient’s tumor.

The original intention of this Special Issue was to provide valuable information on anti-cancer drug development to inspire further research efforts. Fortunately, the research and review articles included in this issue not only achieve this goal but also provide valuable insights into the current state of cancer treatment and highlight potential strategies for improving patient outcomes. We hope that this Special Issue will inspire further research toward the goal of developing more effective and personalized treatments for patients with the life-threatening disease.
